# Impact of paternal support interventions on exclusive breastfeeding and breastfeeding self-efficacy: a systematic review and meta-analysis

**DOI:** 10.1186/s13006-026-00833-w

**Published:** 2026-03-24

**Authors:** Zulfia Samiun, Muhammad Syafar, Citrakesumasari Citrakesumasari, Yahya Thamrin, Apik Indarty Moedjiono, Kadek Ayu Erika, Abdul Salam, Musliha Mustary

**Affiliations:** 1 Doctoral Study Program of Public Health Sciences, Faculty of Public Health, Hasanuddin University, Makassar, Indonesia; 2 Department of Nursing, Faculty of Medicine and Health Science, Muhammadiyah University of Makassar, Makassar, Indonesia; 3 Department of Health Promotion and Behavioral Science, Faculty of Public Health, Hasanuddin University, Makassar, Indonesia; 4 Department of Nutritional Sciences, Faculty of Public Health, Hasanuddin University, Makassar, Indonesia; 5 Department of Occupational Health and Safety, Faculty of Public Health, Hasanuddin University, Makassar, Indonesia; 6 Department of Reproductive Health, Faculty of Public Health, Universitas Hasanuddin, Makassar, Indonesia; 7 Department of Pediatric Nursing, Faculty of Nursing, Hasanuddin University, Makassar, Indonesia; 8 Department of Midwifery, STIKES Salewangang Maros, Maros Regency, Indonesia

**Keywords:** Breastfeeding, Father involvement, Breastfeeding self-efficacy, Support intervention, Systematic review, Meta-analysis

## Abstract

**Background:**

Father support is an important determinant of breastfeeding success; however, evidence regarding its effects on exclusive breastfeeding (EBF) duration and maternal breastfeeding self-efficacy remains limited. This systematic review and meta-analysis evaluated the effectiveness of paternal support interventions on these outcomes.

**Methods:**

This review followed PRISMA 2020 guidelines. Searches were conducted in Scopus, Web of Science, PubMed, and Cochrane Library for studies published between January 2010 and October 2025. Eligible studies included randomized controlled trials or quasi-experimental designs involving postpartum mothers and fathers, father-focused support interventions, standard care comparators, and outcomes related to EBF duration or breastfeeding self-efficacy. Risk of bias was assessed using the Cochrane Handbook. Random-effects models were applied using risk ratios and standardized mean differences.

**Results:**

Six studies involving 716 participants were included. Paternal support interventions increased early breastfeeding initiation by 17% (RR = 1.17; 95% CI: 1.02–1.34), which was not statistically significant at 1 month (RR = 1.30; 95% CI: 0.94–1.81), at four months it was 2.67 times (RR = 2.67; 95% CI: 1.29–5.52), and the effect at six months postpartum did not reach statistical significance (RR = 1.53; 95% CI: 0.99–2.38). Overall breastfeeding success increased by 41% (RR = 1.41; 95% CI: 1.17–1.69). Breastfeeding self-efficacy showed a positive trend compared with usual care (SMD = 1.06; 95% CI: −0.12 to 2.24; *P* = 0.08).

**Conclusion:**

Paternal support interventions are associated with improved early initiation and exclusive breastfeeding duration at four months and showed a positive trend toward increased maternal self-efficacy.

**Supplementary Information:**

The online version contains supplementary material available at 10.1186/s13006-026-00833-w.

## Introduction

Exclusive breastfeeding (EBF) for the first six months of life is strongly recommended by the World Health Organization (WHO) because of its substantial benefits for infant and maternal health, including enhanced immunity, reduced morbidity and mortality, and optimal growth and development [1–3]. Despite these well-documented advantages, EBF rates in many countries, particularly in low- and middle-income settings, remain below global targets [4, 5]. This persistent gap underscores the need for more comprehensive and sustainable breastfeeding promotion strategies.

Breastfeeding support efforts have traditionally focused primarily on mothers. However, increasing evidence indicates that breastfeeding success is shaped not only by maternal knowledge and skills but also by the broader family and social environment, especially the role of fathers [6–8]. Many programs that concentrate exclusively on mothers without actively involving fathers may limit their overall effectiveness. Studies have shown that father-focused education and counseling can improve exclusive breastfeeding rates and breastfeeding continuation [9, 10], and community-based approaches involving fathers have demonstrated stronger effects than mother-only strategies [10–12]. Conversely, when breastfeeding responsibility rests solely on the mother without adequate paternal support, mothers may experience fatigue, stress, and decreased confidence, increasing the risk of early breastfeeding cessation [13]. Qualitative evidence from Indonesia further highlights this issue, identifying inadequate paternal social support as one of the key barriers to achieving exclusive breastfeeding, underscoring the contextual and sociocultural dimensions of father involvement in breastfeeding practices [14].

One important pathway through which paternal involvement may influence breastfeeding outcomes is maternal breastfeeding self-efficacy (BSE). BSE refers to a mother’s confidence in her ability to breastfeed successfully and is a well-established predictor of breastfeeding initiation, duration, and exclusivity [15, 16]. Higher BSE scores are consistently associated with longer breastfeeding continuation and greater likelihood of maintaining EBF [16]. Fathers contribute to strengthening maternal self-efficacy through emotional encouragement, practical assistance, shared caregiving responsibilities, and reinforcement of breastfeeding goals [17]. Counseling and educational programs that actively involve fathers have been shown to enhance both breastfeeding practices and maternal confidence [9, 18].

Although paternal involvement in breastfeeding has received increasing attention, existing studies frequently assess clinical outcomes, such as exclusive breastfeeding duration or initiation, separately from psychosocial determinants, including maternal self-efficacy. Furthermore, the methodological quality of available studies varies, with a limited number of randomized controlled trials and substantial heterogeneity in intervention approaches and follow-up periods. This separation of clinical and psychosocial perspectives limits a comprehensive understanding of how paternal support operates within family-centered breastfeeding dynamics.

To address this gap, the present systematic review and meta-analysis simultaneously evaluates two interrelated outcomes: the duration of exclusive breastfeeding as a clinical indicator and maternal breastfeeding self-efficacy as a key psychosocial determinant. By synthesizing evidence from randomized and quasi-experimental studies conducted across diverse countries and healthcare contexts, this study provides an integrated assessment of the effectiveness of father-focused breastfeeding support strategies. The findings are expected to inform the development of family-centered maternal and child health policies that recognize fathers as essential partners in breastfeeding success.

## Methods

### Protocol and registration

This systematic review and meta-analysis was conducted following the Preferred Reporting Items for Systematic Reviews and Meta-Analyses (PRISMA) statement [19]. The review protocol was not registered in PROSPERO or any other protocol registry. All steps of the review, from formulating the review question, search strategy, study selection, and data extraction to quality assessment, were documented to ensure transparency and reproducibility. The Population Intervention Comparator Outcome Study Design (PICOS) criteria were used to formulate review question systematically and determine inclusion and exclusion criteria to ensure that the selected articles were truly relevant to the research objectives [20]. The PICOS can be seen in Table 1.

**Table 1:** Population intervention comparator outcome study design

*P* – Population	The population included postpartum biological mothers and their male partners identified as fathers or co-parents in the primary studies. No restrictions were applied regarding parental age, marital status, or social status, provided that the father or male partner participated in the breastfeeding support intervention. Cultural background was not used as an eligibility criterion, and studies from diverse sociocultural contexts were eligible. Gender identity was considered as defined in the original studies; none of the included studies explicitly reported the inclusion of transgender or same-sex couples.
I – Intervention	Interventions included father-focused or couple-based breastfeeding support programs in which fathers were the primary target of education, counseling, or engagement strategies aimed at enhancing their supportive role in breastfeeding. These could include educational sessions, counseling, face-to-face training, digital or technology-based programs, written materials, or structured support initiatives designed to strengthen paternal involvement in supporting maternal breastfeeding.
C – Comparator	The comparison group consisted of standard or usual care without breastfeeding support components focused on fathers.
O – Outcome	The primary outcomes were the duration of exclusive breastfeeding at specific postpartum time points and mothers’ self-efficacy regarding breastfeeding. Secondary outcomes included early initiation of breastfeeding and knowledge or attitudes related to breastfeeding when reported.
S – Study design	Randomized controlled trials and quasi-experimental studies were included. Mixed-methods studies were eligible if they contained a quantitative component meeting the inclusion criteria and provided extractable data for effect size calculation. Qualitative findings from mixed-methods studies were not included in the meta-analysis.

### Eligibility criteria

The inclusion criteria are as follows: (1) fathers and mothers without medical conditions that may interfere with breastfeeding (infants are considered ineligible if they are born prematurely, have congenital malformations, chronic illnesses, or medical conditions that require special feeding or contraindicate breastfeeding); (2) the intervention group received father-focused or couple-based breastfeeding support in addition to standard or usual care, while the control group received standard or usual care alone; (3) articles in English; (4) studies assessing the duration of EBF and self-efficacy; (5) randomized controlled trial (RCT)/quasi-experimental design; and (6) data that can be extracted for effect size calculations (e.g., mean and standard deviation).

The exclusion criteria were as follows: (1) mothers or infants with medical conditions that prevented breastfeeding (e.g., HIV, severe mastitis, infants born preterm (< 37 weeks of gestation), those with congenital malformations, chronic illnesses, or medical conditions requiring special feeding or contraindicating breastfeeding were excluded); (2) qualitative studies without quantitative data; (3) articles without full text; (4) studies without a control group; (5) review articles, editorials, or case reports; and (6) duplicate publications.

### Search strategy

Literature searches were performed via the Scopus, Web of Science (WoS), Cochrane Library, and PubMed databases. The literature search was limited from January 2010, to 10 October 2025. The restriction to studies published from 2010 onward reflects the substantial development of theory-informed, father-inclusive, and digitally supported breastfeeding programs over the past decade [8, 21–23]. Limiting the timeframe ensures that the synthesized evidence aligns with contemporary family-centered care models and current health system contexts. All relevant literature was systematically analyzed. The search was restricted to articles published in English due to feasibility considerations and to ensure consistency in data extraction and interpretation.

The article search strategy was as follows: (“partner support” OR “spousal support” OR “paternal support” OR “father support” OR “husband support” OR “male involvement” OR “coparenting”) AND (“breastfeeding” OR “lactation” [MeSH] OR “exclusive breastfeeding” OR “breast feeding” [MeSH]) AND (“self-efficacy” [MeSH] OR “maternal confidence” OR “breastfeeding duration” OR “continuation”)). These keywords were used in all the databases without publication location restrictions. The search strategy was developed collaboratively by three authors (ZS, CC, and MM) and executed independently by the same authors. Title and abstract screening was performed independently by three reviewers (ZS, CC, and YT), followed by full-text assessment against the predefined eligibility criteria.

### Study selection

All the search results were saved, and data screening was performed via Rayyan Intelligent Systematic Review software (https://www.rayyan.ai/). Duplicates were removed, and three authors (ZS, CC, and YT) screened the titles and abstracts. Relevant articles were extracted, and the findings were cross-verified. In addition to peer-reviewed journal articles, grey literature, including theses and dissertations, was also considered for inclusion in order to capture relevant evidence that may not have been published in academic journals.

### Data extraction

Three reviewers (AIM, KAE, and AS) independently extracted the data. Discrepancies were resolved through consensus agreement via MS. No authors of the original studies were contacted for additional clarification. Excluded articles were recorded along with the reasons for exclusion. Data were extracted on the basis of (1) study characteristics (author, year, country, design, and sample size); (2) participant characteristics; (3) details of the intervention and comparator; (4) primary outcomes (EBF, BSE, mean, and standard deviation); and (5) follow-up methods and durations.

### Literature quality assessment

The identified literature was rigorously assessed on the basis of the Cochrane Handbook for Systematic Reviews of Interventions [24]. This handbook assesses validity to evaluate the risk of bias on the basis of six specific criteria: (1) random sequence generation; (2) allocation concealment; (3) blinding of participants/personnel; (4) blinding of outcome assessment; (5) incomplete outcome data; (6) selective reporting; and (7) other biases. Evaluators were asked to categorize each criterion as “low risk,” “high risk,” or “unclear.” If the two evaluation results were inconsistent, all researchers needed to discuss and reach an agreement. The results of the risk of bias assessment are presented in the form of a colored bar chart. The bias risk graph was created via RevMan 5.4.1. Publication bias was assessed visually via a funnel plot according to the Cochrane method to assess potential asymmetry. Evidence certainty assessment (GRADE) was not performed. Risk of bias assessments were performed independently by two reviewers (ZS and MS) using the Cochrane Handbook criteria. Disagreements were resolved through group discussion. All authors reviewed and approved the final analysis and interpretation.

### Statistical analysis

Studies that met the criteria were analyzed via RevMan 5.4.1 software to determine the presence of statistical heterogeneity [25]. For the variable of breastfeeding duration, relative risk (RR) and 95% CI were used as measures of effect, with a p value of less than 0.05 indicating statistical significance. Moreover, for the self-efficacy variable assessment, a random effects model was also used because the mean I² revealed significant heterogeneity (I²=98%; *P* < 0.00001), and the effect measure used standardized mean difference (SMD) due to assessment instrument variation. Heterogeneity was evaluated via the I² statistic. A fixed-effects model was used when I² < 50% and *p* > 0.10, whereas a random-effects model was used when I² ≥ 50% or when there was substantial variation between studies. Subgroup analysis was performed on the basis of the follow-up period. Data analysis was performed by two reviewers (ZS and KAE).

## Results

### Search results and selection

**Fig. 1:**
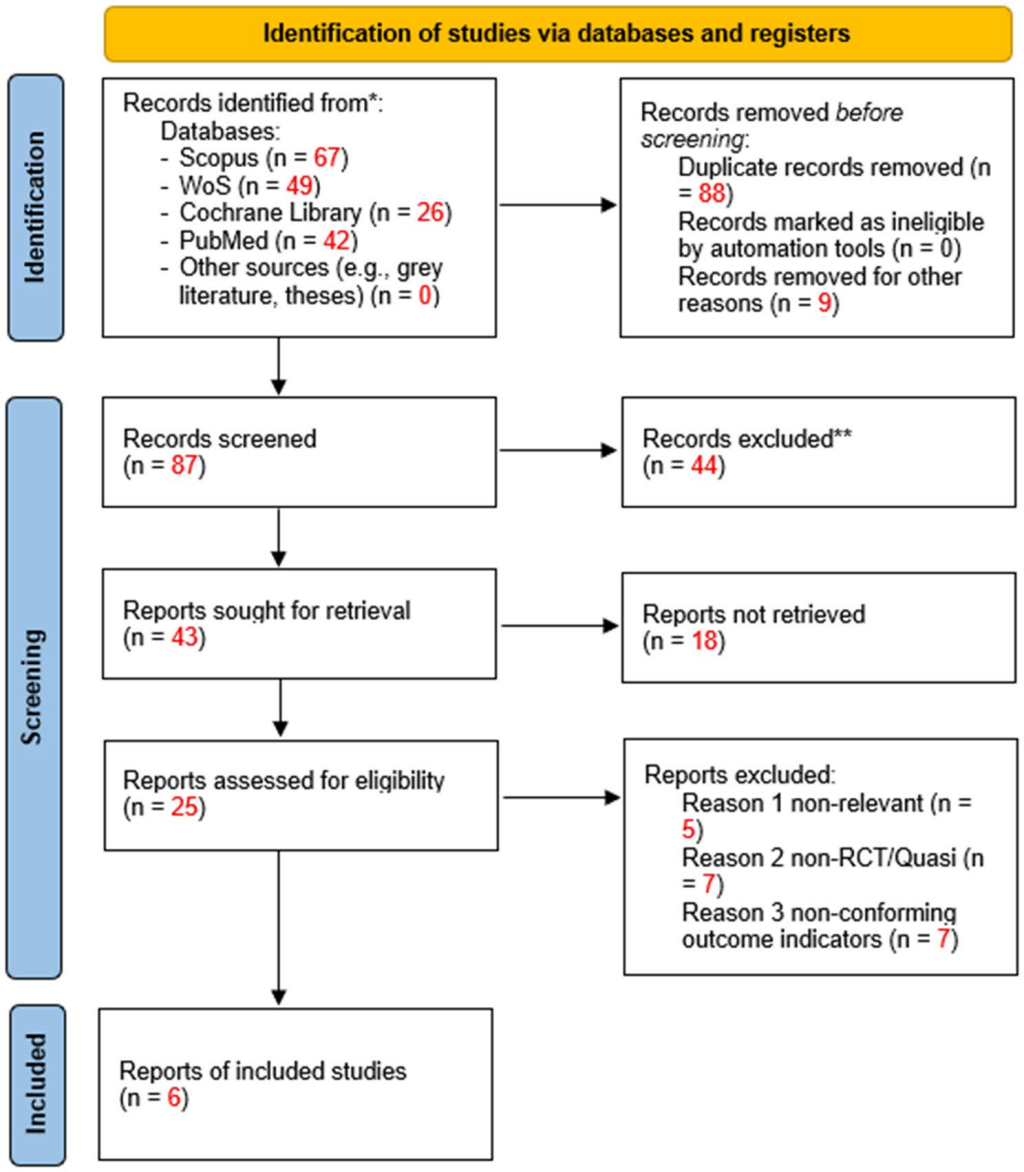
PRISMA flowchart of the study selection process [19]

A total of 184 relevant articles were retrieved, including 67 from Scopus, 49 from Web of Science (WoS), 26 from the Cochrane Library, and 42 from PubMed. After removing duplicate records and screening titles and abstracts, a substantial number of studies were excluded due to irrelevance to the study objectives, non-eligible study designs, or lack of father-focused breastfeeding interventions. In addition, 18 records could not be retrieved because the full texts were unavailable through database access or institutional subscriptions. After screening by the two authors, six articles that met the criteria were ultimately selected. The entire screening process is summarized in the PRISMA diagram (Fig. 1).

### Basic characteristics of the included studies

The six studies [9, 23, 26–29] were conducted across five countries, 1 study from China [23], 2 studies from Canada [28, 29], 1 study from Turkey [26], 1 study from Iran [9], and 1 study from Indonesia [27]. These studies assessed EBF on the basis of the duration of breastfeeding [9, 23, 26–29], and several studies assessed self-efficacy scores [26–29]. The interventions in the studies took the form of verbal breastfeeding education through face-to-face meetings [9, 26, 27, 29], counseling [23, 29], lectures [27], and discussions [27, 29]; the provision of videos or websites [28]; the distribution of booklets or books containing breastfeeding information [26, 29]; and online follow-ups via telephone or WeChat groups [23, 29]. The basic characteristics of all the studies are detailed in Table 2.

**Table 2:** Basic characteristics of the included studies

ID	Author/country/year of publication	Design	*n*	Form of intervention		Main results
				Intervention group	Control group	
1	Abbass-Dick, et al./Canada/2015 [29]	RCT	214 Exp. 107 Cont.107	Receiving multifaceted co-parenting-based breastfeeding support interventions. These interventions include the involvement of both parents in the breastfeeding process, such as discussions at the hospital, co-parenting guidebooks, breastfeeding guidebooks, videos, websites, emails, and phone calls.	Receiving routine care, including standard breastfeeding support in hospitals and any breastfeeding assistance provided proactively in the community.	EBF (1 month and 3 months), self-efficacy score, attitude score, and father’s support score.
2	Abbass-Dick, et al./Canada/2020 [28]	RCT	112 Exp. 56 Cont. 56	Intervention on the eHealth website about breastfeeding based on co-parenting after couples attended a meeting via telephone or web with a research assistant. Couples were shown how to use the website, given a PDF guide containing a description of the content and how to access it, and directed to review the content directly. Couples were also allowed to access additional breastfeeding information resources in the community.	The control group only received information via email stating that they were free to use the public breastfeeding resources available in the community. They were also encouraged to work together as a team in achieving breastfeeding goals and to record the resources they accessed and their level of satisfaction.	EBF (1 month, 3 months, and 6 months), self-efficacy score, knowledge score, attitude score, and father’s support score.
3	Panahi, et al./Iran/2022 [9]	RCT	76 Exp. 38 Cont. 38	Parents attended two face-to-face education and counseling sessions (each lasting approximately 40 min, one week apart) during the second and third weeks of their baby’s life. This intervention was provided individually by a facilitator in a childbirth preparation class.	Only mothers received the standard education provided in the childbirth preparation classes. Four months later, fathers and mothers in both groups were asked to complete a follow-up questionnaire.	Success in early breastfeeding initiation, breastfeeding for 4 months, knowledge score, and father’s support score.
4	Ayran & Çelebioğlu/Turkey/2022 [26]	Quasi-experimental study	97 Exp. 48 Cont. 49	After giving birth, mothers and fathers attend two 40-minute breastfeeding training sessions, which are given individually. In addition, fathers receive an additional 20-minute session focusing on the father’s role in supporting breastfeeding. At the end of the training, fathers are given a breastfeeding education guidebook specifically for fathers.	Parents only receive nursing services that are routinely provided at the hospital.	Early breastfeeding initiation rates and breastfeeding rates (1 month, 2 months, 4 months, and 6 months), and self-efficacy scores.
5	Apri Sulistianingsi, et al./Indonesia/2023 [27]	Quasi-experimental study	138 Exp. 68 Cont. 70	Health education routinely uses the 2020 edition of the Maternal and Child Health (MCH) book. Fathers also receive education to support childbirth. Training is conducted through lectures, discussions, and videos. Training is conducted by health workers, midwives, and doctors. Training is conducted regularly and continuously.	Receiving standard health education on healthy pregnancy, nutrition during pregnancy, preparation for childbirth, signs of labor, and the childbirth process based on the 2020 edition of the Maternal and Child Health (MCH) book.	Success of early breastfeeding initiation, self-efficacy scores, and fathers’ knowledge and skill scores.
6	Huang, et al./China/2024 [23]	RCT	79 Exp. 39 Cont. 40	A 7-session parenting course, a father support group, and individual counseling from late pregnancy to 6 months postpartum. The intervention group was asked to add the researcher to their personal WeChat account, join the WeChat intervention group, and interact with a public WeChat account called “Guardian of Maternal and Infant Health.”	Couples received the standard care available before and after delivery. Researchers contacted mothers by telephone every month.	The rate of EBF from 1 month to 6 months and breastfeeding knowledge scores.

### Results of the meta-analysis and narrative synthesis of secondary results

**Fig. 2:**
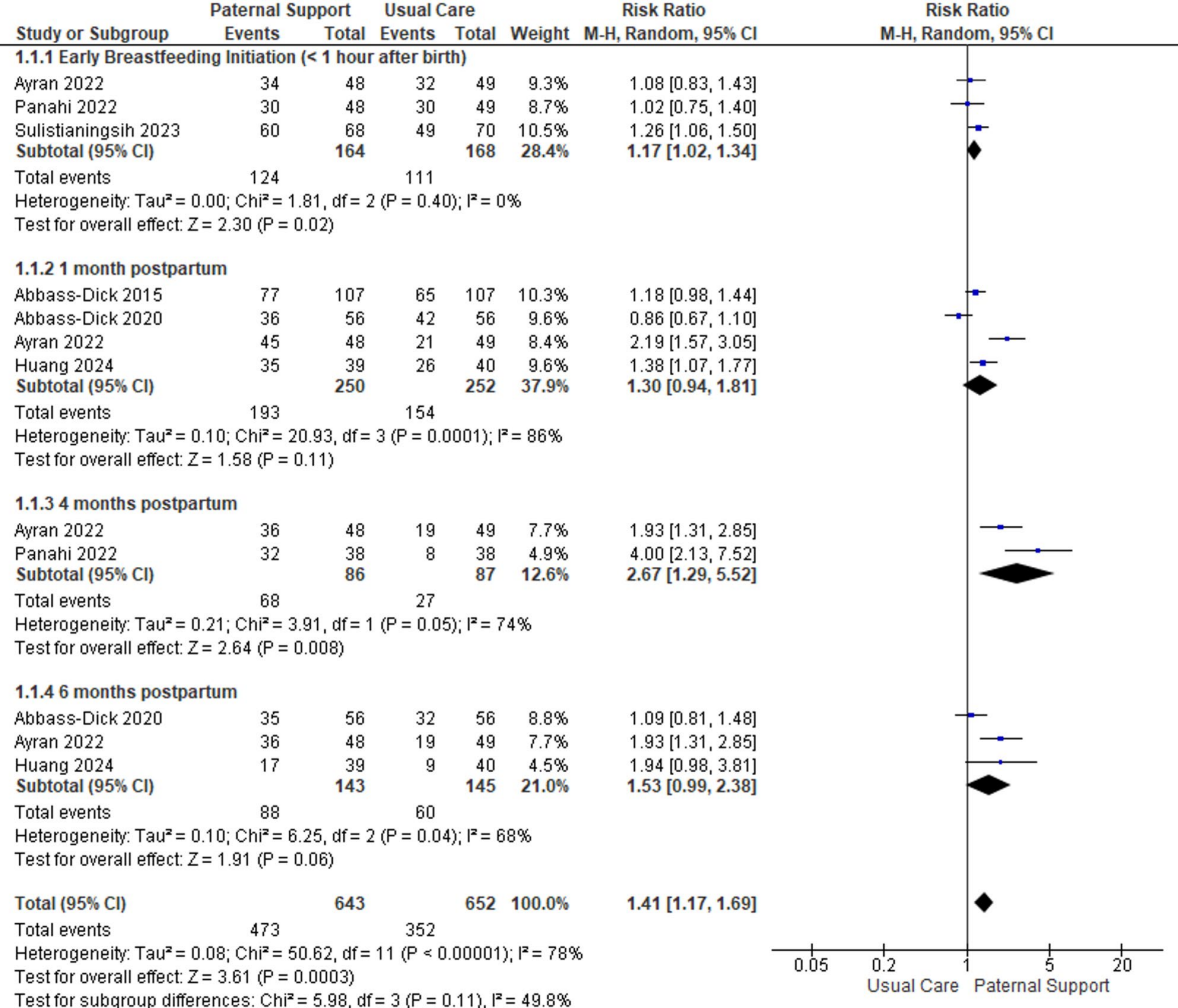
Forest plot of the subgroup effect of paternal support on breastfeeding

The impact of father support interventions on EBF rates at different time points across subgroups is presented in a forest plot (Fig. 2).

A total of six studies [9, 23, 26–29] were included in the analysis, with a total of 716 participants. Substantial heterogeneity was observed among the included studies (I² = 78%; *P* < 0.00001); therefore, a random-effects model was applied for the pooled analysis. Paternal support interventions increased the odds of early breastfeeding initiation (< 1 h after birth) by 17% (RR = 1.17; 95% CI: 1.02–1.34) [9, 26, 27]. At one month postpartum, the pooled effect was not statistically significant (RR = 1.30; 95% CI: 0.94–1.81) [23, 26, 28, 29]. A significant benefit was observed at four months postpartum (RR = 2.67; 95% CI: 1.29–5.52) [9, 26], whereas the effect at six months postpartum did not reach statistical significance (RR = 1.53; 95% CI: 0.99–2.38) [23, 26, 28]. Overall, paternal support increased the likelihood of breastfeeding success by 41% (RR = 1.41; 95% CI: 1.17–1.69). The test for differences between subgroups did not reveal significant variation (*p* = 0.11), suggesting that the effect of paternal support was consistent across different phases of breastfeeding (Fig. 2).

**Fig. 3:**
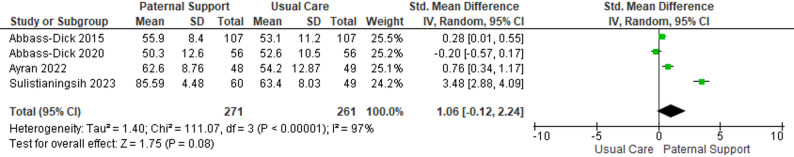
Forest plot of the effect of paternal support on breastfeeding self-efficacy

The meta-analysis of four studies shown in the forest plot (Fig. 3) demonstrated that paternal support interventions were associated with higher maternal breastfeeding self-efficacy scores compared to usual care; however, the overall effect did not reach statistical significance (SMD = 1.06; 95% CI: −0.12 to 2.24; *P* = 0.08) [26–29]. Extremely high heterogeneity was observed among the included studies (I² = 97%), indicating substantial variability in effect sizes. This heterogeneity was likely attributable to differences in the scales used to measure breastfeeding self-efficacy, variations in intervention intensity and duration, and differences in follow-up periods across studies.

Publication bias was evaluated using funnel plots for breastfeeding duration and breastfeeding self-efficacy (Supplementary File 1). The funnel plot for breastfeeding duration (Figure A) showed a generally symmetrical distribution of studies around the pooled relative risk across different postpartum periods, indicating no clear evidence of publication bias. Similarly, the funnel plot for breastfeeding self-efficacy (Figure B) demonstrated an approximately symmetrical pattern around the pooled standardized mean difference, with no obvious small-study effects. Overall, visual inspection of the funnel plots suggests a low risk of publication bias for both outcomes.

Based on the Cochrane Handbook criteria (Supplementary File 2), most studies showed a low risk of bias for random sequence generation (70%) and allocation concealment (50%), although a substantial proportion remained unclear. Blinding of participants and personnel was predominantly rated as high risk (85%), reflecting the nature of the interventions, while blinding of outcome assessment was largely unclear (50%). The majority of studies demonstrated a low risk of bias for incomplete outcome data (85%) and selective reporting (65%). For other sources of bias, only a minority were judged as low risk (15%), with the remainder rated as unclear or high risk.

## Discussion

This systematic review and meta-analysis demonstrated that, compared with standard care, paternal support interventions improved exclusive breastfeeding outcomes and showed a positive trend in enhancing maternal breastfeeding self-efficacy. The strongest effect was observed at four months postpartum. At six months postpartum, the direction of effect remained positive, although it did not reach statistical significance. Overall, paternal support increased the likelihood of breastfeeding success by 41% (RR = 1.41). Regarding maternal breastfeeding self-efficacy, paternal support was associated with higher scores; however, the pooled effect did not reach statistical significance.

Research has shown that fathers’ involvement in interventions significantly increases breastfeeding duration [7, 21, 30]. Studies in Indonesia have revealed that low father support is also associated with reduced likelihood of EBF [31]. The World Breastfeeding Week in August 2025 highlighted the ongoing support that women and babies need from the healthcare system throughout their breastfeeding journey, which means ensuring that every mother has access to support, including support from fathers [32]. Paternal support significantly increases breastfeeding rates [28, 33]. The results from a review of studies in low- and middle-income countries revealed increased early initiation of breastfeeding, EBF, and sustained breastfeeding [34]. Social factors such as family support, cultural norms, and healthcare support have been shown to be important determinants of EBF success; without a supportive environment, mothers often face difficulties in maintaining EBF [13].

Paternal support in the form of paternal intervention significantly increases the likelihood of EBF and prolongs the duration of breastfeeding in the postpartum period compared with groups without paternal support [9, 35]. A recent meta-analysis revealed that interventions involving fathers increase EBF, and paternal support has a positive influence on breastfeeding practices [21]. Effective support from fathers is not only informative but also includes emotional support, practical assistance in caring for the baby and household, and affirmation of the mother, which has been shown to be correlated with increased breastfeeding success [17, 31, 36]. Unlike previous reviews that broadly examined social or family support, this systematic review specifically focuses on support interventions targeted at fathers and their impact on mothers’ breastfeeding self-efficacy and exclusive breastfeeding outcomes. Furthermore, this review synthesizes evidence from randomized and non-randomized studies and provides quantitative estimates through meta-analysis, offering a more comprehensive and focused understanding of the role of father involvement in breastfeeding support.

Interventions that combine lactation education for fathers with counseling, informational media, and direct involvement of fathers in infant care show better results in breastfeeding duration and exclusivity than conventional interventions that target only mothers [34]. The role of fathers as coparents, where they actively participate in lactation education, infant care, and emotional support, has been proven effective in supporting EBF success and maintaining long-term breastfeeding practices [11, 21, 37]. By involving fathers as part of the parenting unit, breastfeeding interventions can strengthen the supportive family environment, increase maternal motivation, and increase the chances of EBF success [7, 8, 12].

Our meta-analysis confirms the beneficial effects of father support interventions on breastfeeding outcomes, which is consistent with previous evidence from recent high-quality reviews. Specifically, the 2024 meta-analytic review by Zhou et al. revealed a consistent increase in EBF rates with inclusive father interventions [21]. Similarly, perinatal interventions involving fathers that include education, counseling, and psychosocial support have been associated with increased initiation, duration, and exclusivity of breastfeeding in various settings [8, 35, 38]. These findings reinforce a paradigm shift toward family-centered maternal and child health strategies, emphasizing that breastfeeding success depends not only on the mother but also on the active involvement of the father.

The high RoB and heterogeneity found in this meta-analysis (see the Risk of Bias and I² sections) reflect significant variability between studies in terms of design, intervention implementation, and context. Some studies reported strong methodological quality, whereas others reported weaknesses in randomization, allocation, or blinding, which are common in behavioral/community intervention studies. This variation may increase the range of effects, explaining why the combined effect remains positive despite high heterogeneity. Nevertheless, the consistent positive results reinforce the belief that paternal involvement provides real benefits, especially when the intervention is implemented with good design and reporting. In addition, analysis of the six-month post-partum subgroup revealed that the effect of the intervention was not statistically significant, although the direction remained positive. These findings differ from those of Mahes et al., who reported that the rate of EBF for up to six months was significantly higher in the intervention group than in the control group [39, 40]. These differences in results may be influenced by variations in the characteristics of the populations included in the studies, such as differences in sociocultural context, health service support, and diverse intervention implementation.

Our findings on BSE underscore the central role of psychological determinants in the success of breastfeeding interventions. Consistent increases in BSE are associated with an increased likelihood of EBF and a longer duration of breastfeeding [12, 41]. According to Dennis (1999), breastfeeding success is strongly influenced by breastfeeding self-efficacy, which is a mother’s belief in her ability to breastfeed, formed through successful experiences, social support, and increased knowledge, so that interventions involving fathers can play an important role in strengthening this belief [15]. Specifically, a study analyzing paternal support in breastfeeding self-efficacy confirmed that higher paternal self-efficacy scores were indeed associated with higher rates of EBF at six weeks post-partum, supporting the idea that fathers play an influential role in breastfeeding dynamics within the family [12]. Interventions that actively involve fathers have been shown to significantly increase mothers’ BSE [22, 42]. Furthermore, findings from studies in various cultural contexts support this assertion. A cohort study in Lebanon and Qatar highlighted that mothers’ attitudes toward breastfeeding are strongly influenced by fathers’ support and knowledge, illustrating how fathers’ involvement can foster a positive breastfeeding environment [43]. Factors that significantly influence fathers’ self-efficacy in supporting breastfeeding include fathers’ knowledge about breastfeeding, fatigue levels, positive emotional support, successful experiences in helping mothers breastfeed, the quality of the marital relationship, and sufficient time spent with the mother and baby [12, 37, 44, 45].

This review has several limitations that should be considered when interpreting the findings. First, substantial heterogeneity between studies indicates variation in population characteristics, intervention delivery, and follow-up duration, which may affect the magnitude of effect estimates. Second, the overall methodological quality was not entirely consistent, with some studies showing unclear or high risk of bias in allocation concealment and blinding, which could reduce internal validity. Third, the number of eligible randomized controlled trials was still limited, and most studies involved relatively small sample sizes, which could affect the statistical precision and generalizability of the results. In addition, the absence of a GRADE assessment means that the certainty of evidence may vary between outcomes. This review also only includes studies published in English, which may have introduced language bias by excluding relevant studies published in other languages. As a result, some evidence from non-English-speaking settings may not have been captured. However, the studies included represent a variety of geographic regions and healthcare contexts, partially mitigating this limitation. Limiting the search to studies published since 2010 may have reduced the number of eligible studies and potentially limited the completeness of the review. Future studies with more rigorous designs and standardized intervention protocols are needed to strengthen the evidence base regarding paternal involvement in breastfeeding support. At the policy level, breastfeeding promotion strategies should consider fathers as key stakeholders and include them in national or institutional breastfeeding guidelines and training curricula for healthcare professionals.

## Conclusion

This systematic review and meta-analysis indicates that father support interventions are associated with improved breastfeeding outcomes, including early initiation and increased likelihood of exclusive breastfeeding at four months. The intervention also showed a positive trend toward increased maternal self-efficacy. Despite heterogeneity across studies and some unclear risk of bias, the overall evidence highlights the important role of paternal involvement in breastfeeding success. These findings support the integration of father-focused breastfeeding support into family-centered perinatal care and maternal and child health policies.

## Supplementary Information

Below is the link to the electronic supplementary material.

Supplementary Material 1

Supplementary Material 2

## Data Availability

No datasets were generated or analysed during the current study. All studies included in this review are published and publicly available.
