# Supplementary material for: Impact of paternal support interventions on exclusive breastfeeding and breastfeeding self-efficacy: a systematic review and meta-analysis
Source: Int Breastfeed J. 2026 Mar 24;21:45. doi: 10.1186/s13006-026-00833-w (PMC13134082; doi:10.1186/s13006-026-00833-w)
Supplement: Supplementary file 1 — Supplementary Material 1 [file 13006_2026_833_MOESM1_ESM.docx]

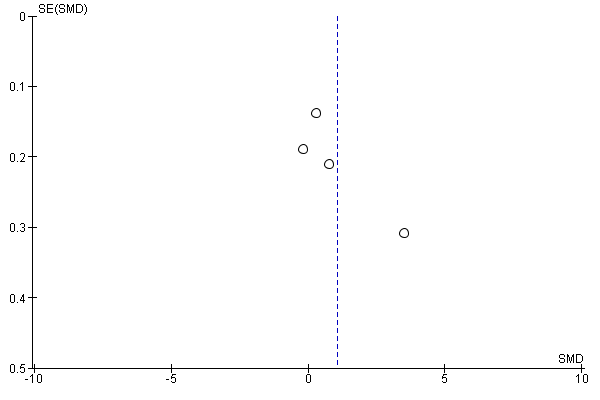

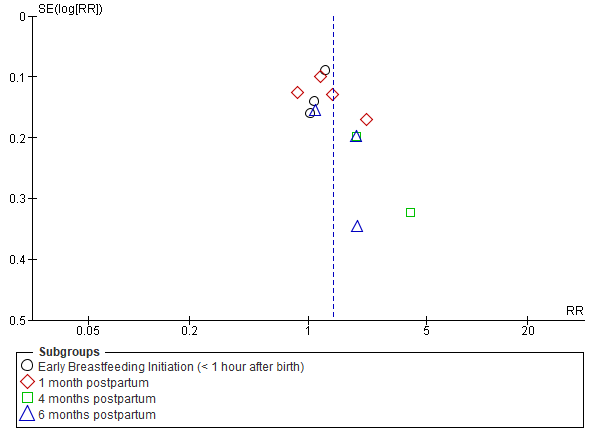
Supplementary File 1

A B

Funnel plot assessing publication bias for the effect of paternal support on breastfeeding duration (figure A) and breastfeeding self-efficacy (figure B)
