# Supplementary figures and images for: Impact of paternal support interventions on exclusive breastfeeding and breastfeeding self-efficacy: a systematic review and meta-analysis
Source: Int Breastfeed J. 2026 Mar 24;21:45. doi: 10.1186/s13006-026-00833-w (PMC13134082; doi:10.1186/s13006-026-00833-w)

Supplementary File 2


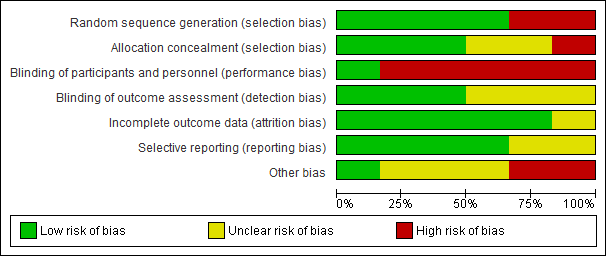


Assessment of bias risk in the included studies

Supplement: Supplementary file 2 — Supplementary Material 2 [file 13006_2026_833_MOESM2_ESM.docx]
